# Impact on Health-Related Quality of Life of Video-Assisted Thoracoscopic Surgery for Lung Cancer

**DOI:** 10.1245/s10434-019-08090-4

**Published:** 2019-12-01

**Authors:** Kerry N. L. Avery, Jane M. Blazeby, Katy A. Chalmers, Timothy J. P. Batchelor, Gianluca Casali, Eveline Internullo, Rakesh Krishnadas, Clare Evans, Doug West

**Affiliations:** 1 Bristol Centre for Surgical Research, University of Bristol, Bristol, UK; 2 Division of Surgery, University Hospitals Bristol NHS Foundation Trust, Bristol, UK

## Abstract

**Background:**

Video-assisted thoracoscopic surgery (VATS) approaches are increasingly used in lung cancer surgery, but little is known about their impact on patients’ health-related quality of life (HRQL). This prospective study measured recovery and HRQL in the year after VATS for non-small cell lung cancer (NSCLC) and explored the feasibility of HRQL data collection in patients undergoing VATS or open lung resection.

**Patients and Methods:**

Consecutive patients referred for surgical assessment (VATS or open surgery) for proven/suspected NSCLC completed HRQL and fatigue assessments before and 1, 3, 6 and 12 months post-surgery. Mean HRQL scores were calculated for patients who underwent VATS (segmental, wedge or lobectomy resection). Paired *t*-tests compared mean HRQL between baseline and expected worst (1 month), early (3 months) and longer-term (12 months) recovery time points.

**Results:**

A total of 92 patients received VATS, and 18 open surgery. Questionnaire response rates were high (pre-surgery 96–100%; follow-up 67–85%). Pre-surgery, VATS patients reported mostly high (good) functional health scores [(European Organisation for Research and Treatment of Cancer) EORTC function scores > 80] and low (mild) symptom scores (EORTC symptom scores < 20). One-month post-surgery, patients reported clinically and statistically significant deterioration in overall health and physical, role and social function (19–36 points), and increased fatigue, pain, dyspnoea, appetite loss and constipation [EORTC 12–26; multidimensional fatigue inventory (MFI-20) 3–5]. HRQL had not fully recovered 12 months post-surgery, with reduced physical, role and social function (10–14) and persistent fatigue and dyspnoea (EORTC 12–22; MFI-20 2.7–3.2).

**Conclusions:**

Lung resection has a considerable detrimental impact on patients’ HRQL that is not fully resolved 12 months post-surgery, despite a VATS approach.

**Graphic Abstract:**

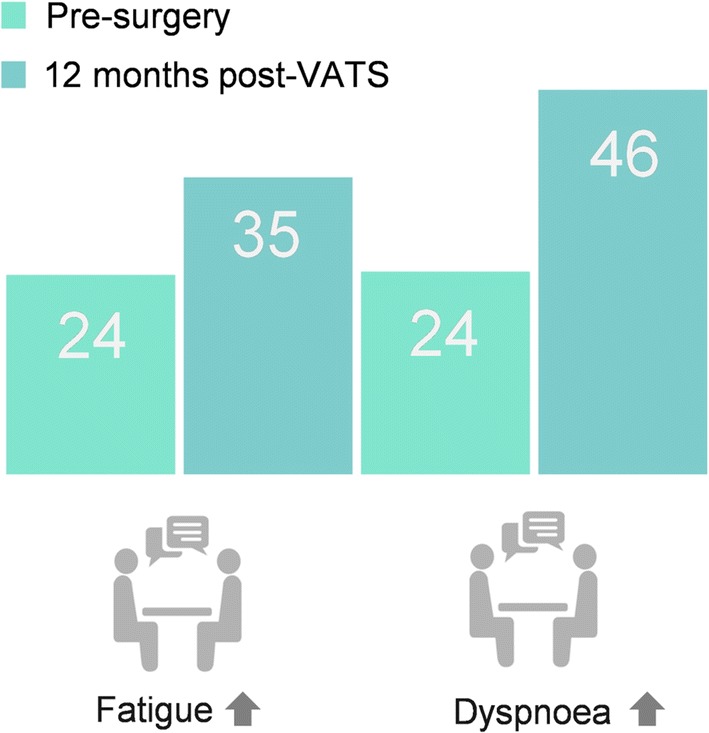

**Electronic supplementary material:**

The online version of this article (10.1245/s10434-019-08090-4) contains supplementary material, which is available to authorized users.

Lung resection is a mainstay of therapy for early-stage lung cancer.^1^ Resection traditionally involves a thoracotomy, which may be associated with significant mortality and morbidity.^2^ Increasingly, video-assisted thoracoscopic surgery (VATS) approaches have been used;^1^ whilst data to support the safety of VATS are available, few well-designed multi-centre studies have compared thoracotomy and VATS surgery, although studies are ongoing.^3^ In addition to understanding mortality and morbidity outcomes of surgery, the need to assess the impact on aspects of patients’ health-related quality of life (HRQL) using validated patient-completed questionnaires has been increasingly acknowledged.^4^

Lung resection has been associated with a significant detrimental impact on patients’ short- to medium-term HRQL, including reduced physical, role and mental function and increased pain, in several prospective observational studies.^5^–^7^ Little is still known, however, about the impact of minimal access lung resection on HRQL, and the few prospective studies available have methodological limitations. Larger studies have explored HRQL after VATS but have been retrospective or cross-sectional in design and do not measure HRQL pre-operatively.^8^,^9^ Prospective studies measuring HRQL before and after VATS surgery have typically been small in size or used unvalidated or generic instruments that measure broad aspects of health, which may not adequately capture the complex and unique areas of function impaired by lung cancer^6^,^10^–^13^ or studied few patients.^11^ Between 2008 and 2014, Bendixen and colleagues^14^ randomised 206 early-stage lung cancer patients to open or thoracoscopic lobectomy in a single centre in Denmark. Patients completed several measures [EuroQol EQ-5D-3L questionnaire, a generic measure of health status; EORTC core quality of life questionnaire (QLQ-C30); pain rating scale] at baseline and at several time points for 12 months post-operatively. However, response rates and data completeness were low, and fatigue, the most common acute symptom reported by patients before and after lung cancer treatment,^15^ was not assessed in detail using a validated measure. It remains that little is known about the impact of minimal access lung resection on patients’ HRQL and whether high-quality self-reported HRQL data can be collected from this patient group. This prospective study measured and described in detail the HRQL of patients before and during the first year after VATS for non-small cell lung cancer (NSCLC). A secondary aim of the present work is to explore the feasibility of collecting self-reported HRQL data in a sample of patients undergoing surgery (VATS or open lung resection) for NSCLC.

## Patients and Methods

A prospective questionnaire cohort study was conducted at a UK academic hospital.

### Patients

From May 2014 to April 2015, men and women aged 18 years or over referred to the thoracic surgery service at University Hospitals Bristol NHS Foundation Trust (UHBT) for surgical assessment for proven or suspected NSCLC were screened for study eligibility at the first surgical consultation following referral. Patients were excluded if they had previous or concurrent malignancies or had insufficient capacity or understanding of English to provide written informed consent.

Routine staging investigations included a computerised tomography (CT) scan of chest and upper abdomen and fluorodeoxyglucose positron emission tomography (FDG-PET) scan. Spirometry and lung carbon monoxide transfer factor were routinely measured pre-operatively, in accordance with British Thoracic Society guidelines.^16^ No patients received adjuvant immunotherapy.

Eligible patients were posted a participant information leaflet (PIL) after notification of referral for surgical assessment for proven or suspected lung cancer and a hospital outpatient appointment for a surgical team consultation, to enable patients time to consider study participation should they choose to proceed with surgery. Patients choosing to proceed with surgery at their outpatient consultation were invited to attend the pre-operative assessment clinic on the same day, where they were approached by the research nurse about study participation. Patients expressing an interest were asked by the research nurse to give written informed consent for the present study. Baseline demography and clinical details were collected, and baseline HRQL questionnaires administered. Ethics committee approval was granted from the West Midlands–Edgbaston Research Ethics Committee, UK.

### Surgery and Peri-operative Care

A team of five consultant thoracic surgeons (T.B., G.C., E.I., R.K. and D.W.) from UHBT performed all surgeries on consenting patients. Thoracoscopic surgery involved single-lung ventilation, using a 10-mm 30° thoracoscopic camera and, usually, a total of three thoracoscopic ports. Lobectomy was performed using an anterior approach described by Hansen and colleagues.^17^ Rib spreading was avoided.

Patients were managed peri-operatively using an institutional enhanced recovery after surgery (ERAS) pathway, which included day-of-surgery admission, avoidance of prolonged fasting, carbohydrate loading, use of minimal access surgery and regional anaesthesia (when possible), single chest drains and early mobilisation after surgery. Patients were given carbohydrate drinks on the morning of surgery (400 ml Nutritcia preOp™; Trowbridge, UK), then daily supplementary drinks until discharge (Fortisips, Nutricia; Trowbridge, UK). Early mobilisation was encouraged post-operatively. Post-operative chemotherapy was offered to patients with good performance status with node involvement or tumours > 4 cm diameter.

Consenting patients who subsequently opted out of surgery or whose diagnosis changed prior to surgery were excluded. Patients whose surgery was converted from VATS to open were excluded as it was hypothesised that HRQL in converted patients was likely to approximate that of planned open surgery cases. Patients whose diagnosis changed (e.g. to a benign diagnosis) following pathological assessment were also excluded as it was hypothesised that surgery for benign conditions may affect HRQL differently from those with NSCLC.

### Demographic and Clinical Characteristics of Participants

Demographic, clinical and operative details of eligible participants undergoing VATS or open surgery were collected, tabulated and analysed using descriptive statistics.

### Assessment of Health-Related Quality of Life in Patients Undergoing Video-Assisted Thoracoscopic Surgery Lung Resection

HRQL was assessed using two validated questionnaires: EORTC QLQ-C30 (generic) (version 3.0)^18^ and EORTC QLQ-LC13 (lung cancer module).^19^ The core questionnaire assesses generic aspects of health, including physical, emotional and social function and symptoms that commonly occur in patients with cancer. The lung cancer module assesses specific issues related to this group of patients, including breathlessness, appetite loss and cough, comprising one symptom scale (dyspnoea) and ten single items (coughing, haemoptysis, sore mouth, dysphagia, peripheral neuropathy, alopecia, pain in chest, pain on arm or shoulder, pain in other parts and pain medication).

EORTC responses were rated on a four-point Likert scale and transformed linearly to give scores from 0 to 100. In function scales with multiple items, higher scores indicate a higher level of functioning, while higher scores on symptom scales and single items indicate more symptoms. A five-to-ten-point or greater change in score is considered clinically significant.^20^ The validated multidimensional fatigue inventory MFI-20^21^ was used to assess fatigue in detail, as this is the most common acute symptom reported by patients before and after lung cancer treatment.^15^ The MFI-20 comprises five dimensions (general fatigue, physical fatigue, reduced activity, reduced motivation and mental fatigue). Each dimension includes four items, two that indicate fatigue and two that are contradictory of fatigue, rated on a five-point Likert scale. Scores for the contradictory items were inverted, and a cumulative score for each dimension was calculated. Scores for each dimension ranged from 4 to 20. Higher scores for general, physical and mental fatigue indicate worse fatigue, whilst higher scores for reduced activity and reduced motivation indicate greater reduced activity and motivation. A change in score of ≥ 2 points is considered clinically relevant.^22^ A qualitative descriptive system (e.g. “good”, “moderate” and “poor”) has not yet been developed for the interpretation of EORTC scale scores.^23^ Reference scores for the patient population included in the present study are not yet available.^23^ EORTC scores have therefore been interpreted considering data from other available published studies.^23^,^24^

HRQL assessment points were selected to enable changes in participants’ HRQL and recovery to be described. Participants were asked to complete the first (baseline) set of questionnaires at their pre-operative assessment clinic, within 1 month prior to surgery. Participants were then posted questionnaires at 1, 3, 6 and 12 months post-surgery. Patients who did not return a questionnaire received one telephone reminder approximately 3–4 weeks after the questionnaire was due.

### Data Analyses

Questionnaire response rates and reasons for non-completion were examined using descriptive statistics. Assessment of the impact on HRQL and recovery from either VATS or open surgery was planned a priori. However, the number of eligible participants receiving open surgery was too small to enable accurate assessment of recovery and impact on HRQL. HRQL analyses were therefore conducted for patients undergoing VATS only. Mean HRQL scores, standard deviations and 99% confidence intervals were calculated for QLQ-C30, QLQ-LC13 and MFI-20 scales and/or single items at all time points to describe recovery and the impact of surgery on HRQL during the first year post-VATS. Paired *t*-tests were performed post hoc (after seeing the data) to explore comparisons between baseline HRQL scores and those at expected worst (1 month), early (3 month) and longer-term (12 month) recovery time points for scales and items where changes were considered clinically relevant (≥ 10-point change in EORTC scores; ≥ 2-point change in MFI-20 scores).^20^,^22^*t*-Tests were not performed for the 6-month time point to minimise the number of statistical tests performed, reducing the probability of a type I error (false-positive finding). A significance criterion of 1% was used throughout. Missing data were imputed according to the EORTC guidelines.^25^ All analyses were performed using Stata statistical software version 14.2 (StataCorp, USA).

This study is reported in accordance with the Strengthening the Reporting of Observational Studies in Epidemiology (STROBE) guidelines.^26^

## Results

**Fig. 1:**
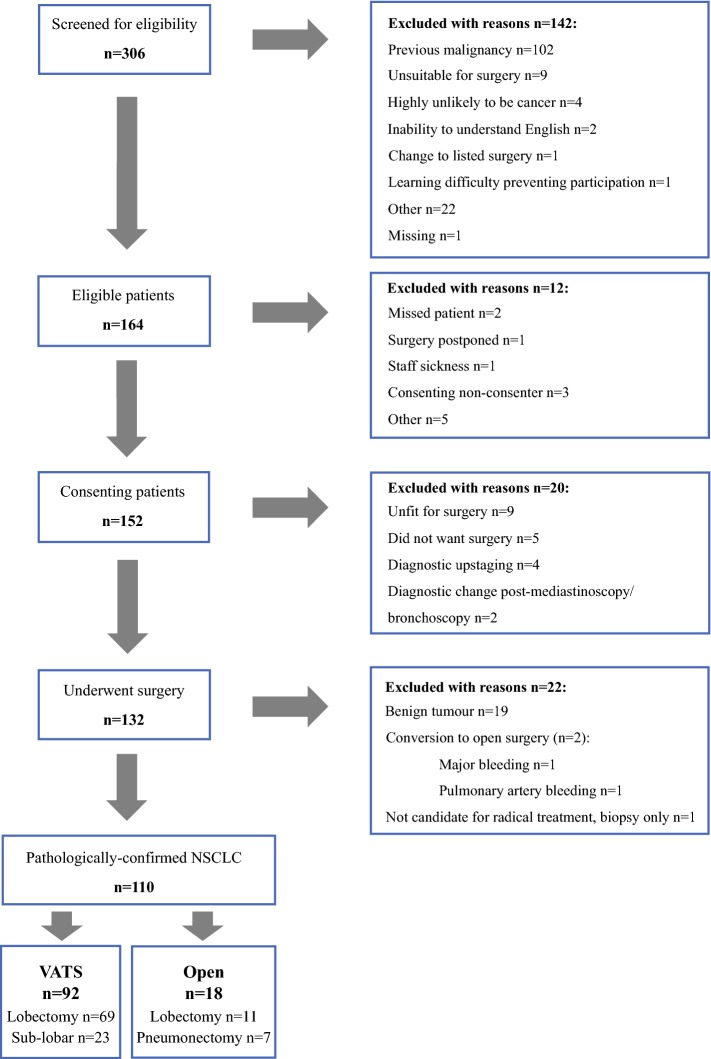
Flow diagram illustrating eligibility screening through to surgery for patient with pathologically confirmed NSCLC

From the 306 patients screened, 164 (54%) were eligible (Fig. 1); 12 (7.3%) were not enrolled for reasons specified in Fig. 1, including 5 (3.0%) patients who declined to take part. No further details were collected on these patients. From the 152 (93%) patients who consented to participate, 131 (86%) went on to have surgery; 112 (85%) and 19 (15%) were planned for VATS and open surgery, respectively, but 2 were converted from VATS to open resection during surgery and were subsequently excluded. A further 19 patients whose pathological assessment confirmed benign lesions were excluded. Therefore, 110 patients were included in the final analyses [92 (84%) VATS and 18 (16%) open surgery].

### Patient Baseline Demographic and Clinical Characteristics

Baseline (pre-surgery) demographic and clinical details of the 110 patients who underwent VATS or open surgery are presented in Supplementary Table S1. Patients selected for VATS and open surgery were similar in terms of sex, but appeared to be different in other characteristics: VATS patients were older (mean age 70 vs 65 years), more often diagnosed pre-operatively with lower-stage tumours (IA, IB or IIA 72% vs 33%) and had a better thoracic surgery scoring system score (Thoracoscore) [median interquartile range (IQR) 1.5 (1.2, 1.9) vs 2.4 (1.8, 4.5)] than those selected for open surgery. In addition, patients selected for VATS were less likely to be obese [body mass index (BMI) ≥ 30 kg/m^2^] (33% vs 56%) and more likely to be current smokers (21% vs 0%).

### Patient Peri- and Post-operative Details

Details of the surgical procedure performed and peri- and post-operative details are provided in Supplementary Table S2. Longer-term (12-month post-operative) clinical outcomes are detailed in Supplementary Table S3. At the end of the 12-month follow-up period, fewer patients undergoing VATS compared with those undergoing open surgery had received further treatment for cancer (29% vs 56%), and fewer VATS patients had died (11% vs 33%).

### Questionnaire Completion Rates and Reasons for Withdrawal

Questionnaire response rates at each time point and reasons for non-completion were included in the analyses (Table 1). In total, 106/110 (96%) patients completed HRQL questionnaires pre-surgery, and questionnaire response rates during follow-up were high, ranging from 67 to 85% at each time point.

**Table 1:** EORTC QLQ-C30 and LC-13 questionnaire response rates and reasons for non-completion for consenting patients undergoing VATS or open surgery (*n* = 110)

	Baseline			1 month post-surgery			3 months post-surgery			6 months post-surgery			12 months post-surgery		
	VATS	Open	All	VATS	Open	All	VATS	Open	All	VATS	Open	All	VATS	Open	All
Eligible patients	92	18	110	91^a^	18	109^a^	89^b^	16^c^	105^d^	87^e^	15^f^	102^g^	82^h^	12^i^	94^j^
Returned questionnaires (%)	88 (95.7)	18 (100)	106 (96.3)	71 (78.0)	12 (66.7)	83 (76.1)	67 (75.3)	12 (75.0)	79 (75.2)	74 (85.1)	12 (80.0)	86 (84.3)	62 (75.6)	10 (83.3)	72 (76.6)
Questionnaire not sent due to patient withdrawal from study	0	0	0	2	0	2	5	1	6	7	1	8	9	1	10
Questionnaire not sent due to administrative error	0	0	0	1	0	1	2	0	2	0	0	0	0	0	0
Not returned for unknown reason	2	0	2	13	4	17	13	0	13	4	0	4	8	0	8
Patient withdrew from study after receiving the questionnaire	1	0	1	2	1	3	2	0	2	0	0	0	0	0	0
Patient too unwell to complete or died after questionnaire was sent	1	0	1	2	1	3	0	3	3	2	2	4	3	1	4

*VATS* video-assisted thoracoscopic surgery

^a^One VATS patient died between surgery and 1-month follow-up

^b^Three VATS patients died between surgery and 3-month follow-up (two VATS patients died within 28 days of surgery but completed and returned their 1 month follow-up questionnaire)

^c^Two open patients died between surgery and 3-month follow-up

^d^Five patients died between surgery and 3-month follow-up

^e^Five VATS patients died between surgery and 6-month follow-up

^f^Three open patients died between surgery and 6-month follow-up

^g^Eight patients died between surgery and 6-month follow-up

^h^Ten VATS patients died between surgery and 12-month follow-up

^i^Six open patients died between surgery and 12-month follow-up

^j^16 patients died between surgery and 12-month follow-up

### Health-Related Quality of Life Before Video-Assisted Thoracoscopic Surgery Lung Resection

**Fig. 2:**
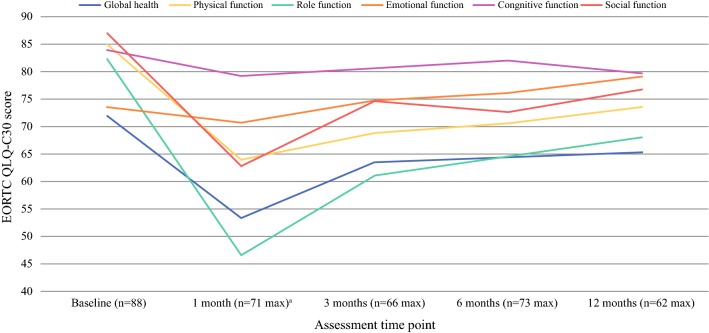
Mean EORTC QLQ-C30 function scores for patients undergoing VATS. Higher scores for measures of function (global health–social function) suggest a higher level of function. A variable that scored at least 10 points greater or less than the baseline score is considered clinically relevant

**Fig. 3:**
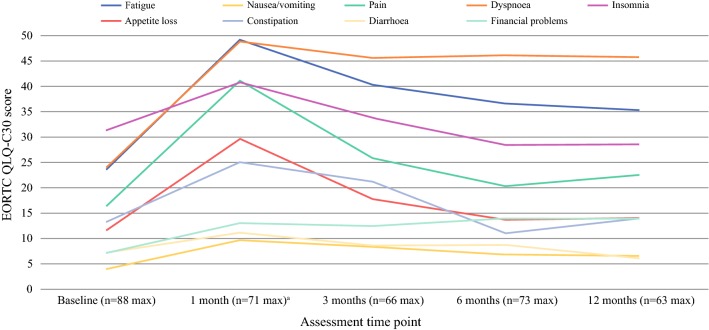
Mean EORTC QLQ-C30 symptom scores for patients undergoing VATS. Higher scores for symptom scales/items (fatigue–pain other) suggest an increased effect of these symptoms on patients. A variable that scored at least 10 points greater or less than the baseline score is considered clinically relevant

Before VATS (baseline), patients reported high function scores indicating good overall (global) health and good physical, role, cognitive and social function, though lower levels of emotional function (Table 2; Fig. 2). Patients also reported marked insomnia and fatigue, and mild dyspnoea, appetite loss and constipation (Table 2; Fig. 3).

**Table 2:** Means and 99% confidence intervals for EORTC QLQ-C30, EORTC QLQ-LC13 and MFI-20 questionnaire scores at each assessment time point for patients undergoing VATS

Variable	Baseline			1 month post-surgery			3 months post-surgery			6 months post-surgery			12 months post-surgery		
	*N*^a^	Mean	99% CI^b^	*N*	Mean	99% CI	*N*	Mean	99% CI	*N*	Mean	99% CI	*N*	Mean	99% CI
QLQ-C30															
Global health and functional scales^c^															
Global health	88	71.9	66.3, 77.5	64	**53.3**	**45.7, 60.8**	63	63.5	58.3, 68.7	71	63	57.4, 68.7	60	65.3	59.3, 71.2
Physical function	88	84.9	80.0, 89.8	71	**63.9**	**57, 70.8**	66	**68.8**	**62.3, 75.3**	73	**68.9**	**62.4, 75.4**	62	**73.5**	**67.3, 79.7**
Role function	88	82.2	74.4, 90.0	71	**46.5**	**36.3, 56.7**	66	**61.1**	**52.4, 69.8**	73	**62.8**	**53.8, 71.8**	62	**68**	**58.4, 77.6**
Emotional function	88	73.5	67.4, 79.6	64	70.7	62.3, 79.1	63	74.7	67.1, 82.4	71	76	69.0, 83.0	60	79	71.9, 86.2
Cognitive function	88	83.9	79.0, 88.8	64	79.2	71.6, 86.7	63	80.7	74.7, 86.6	71	81.9	75.3, 88.6	60	79.7	72.3, 87.2
Social function	88	86.9	80.5, 93.4	64	**62.8**	**52.2, 73.3**	63	**74.6**	**66.3, 82.9**	71	**71.1**	**61.6, 80.7**	60	**76.7**	**67.1, 86.2**
Symptom scales/items^d^															
Fatigue	88	23.7	17.6, 29.9	70	**49.2**	**41.3, 57.1**	66	**40.3**	**33.5, 47.2**	73	**39.1**	**31.7, 46.6**	62	**35.3**	**27.3, 43.3**
Nausea/vomiting	88	4.0	1.1, 6.8	71	9.6	3.6, 15.6	66	8.3	3.0, 13.7	72	6.9	2.1, 11.8	62	6.5	13., 11.6
Pain	88	16.5	9.4, 23.5	71	**41.1**	**31.6, 50.6**	66	25.8	16.4, 35.1	73	23.5	15.4, 31.7	63	22.5	13.3, 31.7
Dyspnoea	87	24.1	17.6, 30.7	71	**48.8**	**39.3, 58.3**	65	**45.6**	**36.6, 54.7**	71	**47**	**38.2, 55.7**	62	**45.7**	**35.5, 55.9**
Insomnia	87	31.4	21.5, 41.3	71	40.8	29.2, 52.5	66	33.8	23.1, 44.6	73	30.1	20.4, 39.9	62	28.5	17.9, 39.1
Appetite loss	88	11.7	6.6, 16.9	71	**29.6**	**18.6, 40.6**	66	17.7	9.3, 26.0	72	11.6	4.8, 18.4	62	14	6.5, 21.5
Constipation	88	13.3	6.1, 20.4	64	**25**	**13.5, 36.5**	63	21.2	11.3, 31.0	71	13.6	6.2, 21.0	60	13.9	6.2, 21.6
Diarrhoea	88	7.2	1.6, 12.8	63	11.1	2.4, 19.8	62	8.6	1.9, 15.3	71	8.9	2.87, 15.0	60	6.1	1.2, 11.1
Financial problems	88	7.2	2.2, 12.2	64	13	4.1, 22.0	62	12.4	4.7, 20.1	70	15.2	7.1, 23.4	60	13.9	5.4, 22.4
QLQ-LC13															
Symptom scales/items^d^															
Dyspnoea	86	18.7	13.9, 23.6	63	**36.2**	**27.6, 44.7**	63	**33.7**	**26.2, 41.2**	68	**34.6**	**26.7, 42.6**	62	**33.9**	**25.4, 42.4**
Coughing	87	35.2	28.6, 41.9	70	37.6	30.5, 44.8	66	34.9	27.9, 41.9	72	36.1	28.4, 43.9	63	34.4	25.4, 43.4
Haemoptysis	86	3.5	0.2, 6.8	70	7.6	1.9, 13.4	67	1	-0.9, 2.9	72	1.9	-0.6, 4.3	63	0	0.0, 0.0
Sore mouth	86	5.4	1.4, 9.5	70	10.5	2.5, 18.4	67	7.5	1.9, 13.1	72	5.1	1.3, 8.9	63	7.4	1.9, 12.9
Dysphagia	87	3.8	0.5, 7.2	70	10.5	3.0, 18.0	67	8.5	1.6, 15.3	71	6.1	1.0, 11.2	63	6.4	1.1, 11.6
Peripheral neuropathy	87	11.1	5.3, 17.0	70	10	4.0, 16.1	65	9.7	2.8, 16.7	70	15.7	8.4, 23.1	62	19.4	11.1, 27.7
Alopecia	87	4.2	0.8, 7.7	68	1.5	-0.8, 3.7	66	4.6	0.3, 8.8	72	10.7	3.7, 17.6	62	3.8	-0.8, 8.4
Pain in chest	87	10.7	5.7, 15.8	70	**32.9**	**23.8, 41.9**	66	**21.7**	**13.7, 29.7**	71	19.3	11.4, 27.1	62	19.4	11.1, 27.7
Pain in arm	86	12.8	6.1, 19.5	69	20.8	11.3, 30.3	66	16.2	7.0, 25.4	71	17.8	9.0, 26.7	63	18	10.5, 25.4
Pain other	84	11.9	7.3, 16.5	67	**28.4**	**24.5, 32.2**	66	18.2	12.7, 23.6	71	15	9.8, 20.3	61	17.5	11.8, 23.2
MFI-20															
Fatigue dimensions^e^															
General fatigue	86	9.7	8.5, 10.9	66	**13.5**	**12.3, 14.7**	64	**13.2**	**11.8, 14.5**	71	**12.4**	**11.0, 13.8**	57	**12.4**	**10.7, 14.0**
Physical fatigue	88	9.5	8.4, 10.6	67	**14.2**	**12.8, 15.6**	65	**13.5**	**12.1, 14.8**	73	**12.6**	**11.2, 14.0**	59	**12.5**	**10.9, 14.1**
Mental fatigue	88	8.2	7.0, 9.3	68	8.9	7.4, 10.3	65	8.5	7.0, 10.1	73	8.8	7.3, 10.3	60	8.9	7.3, 10.5
Reduced activity	87	9	7.3, 10.3	65	**14**	**12.5, 15.6**	66	**13.3**	**11.7, 14.8**	72	**12**	**10.6, 13.5**	57	**12.2**	**10.6, 13.9**
Reduced motivation	84	8.3	7.2, 9.4	66	**11.2**	**9.7, 12.7**	66	10.1	8.8, 11.4	70	9.6	8.4, 10.9	58	10	8.6, 11.3

*CI* confidence interval

^a^Some data missing due to patients not completing all questionnaire items

^b^99% confidence intervals for mean scores

^c^Higher scores for measures of global health and functional scales (physical–social function) indicate better health/function. Bold numbers indicates a change in score from baseline of ≥ 10 points and is considered clinically relevant

^d^Higher scores for symptom scales/items (fatigue–pain other) indicate an increased effect of these symptoms on patients. Bold numbers indicates a change in score from baseline of ≥ 10 points and is considered clinically relevant

^e^Higher scores for general, physical and mental fatigue indicate worse fatigue. Higher scores for reduced activity and reduced motivation indicate greater reduced activity and motivation. Bold numbers indicates a change in score from baseline of ≥ 2 points and is considered clinically relevant

### Health-Related Quality of Life After Video-Assisted Thoracoscopic Surgery Lung Resection

#### Function Scales (EORTC Questionnaires)

One month after VATS, patients’ overall health and physical, role and social function had deteriorated by a clinically meaningful amount (≥ 10 points, 19–36; Table 2; Fig. 2). At 3 months post-surgery, overall health had recovered to pre-surgery levels, but problems with reduced physical, role and social function persisted and were still present 12 months post-surgery, with a reduction in scores from baseline ranging from 10 to 14 points.

#### Symptom Scales (EORTC Questionnaires)

**Fig. 4:**
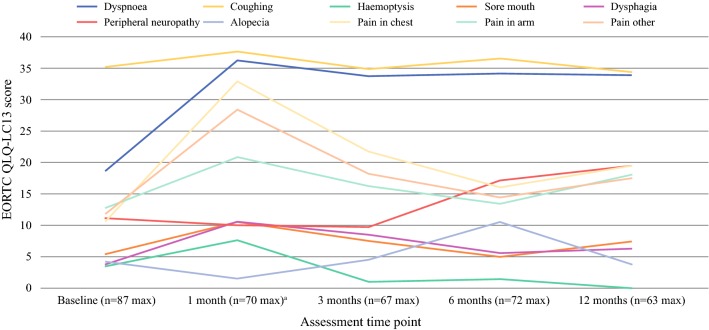
Mean EORTC QLQ-LC13 symptom scores for patients undergoing VATS. Higher scores for symptom scales/items (fatigue–pain other) suggest an increased effect of these symptoms on patients. A variable that scored at least 10 points greater or less than the baseline score is considered clinically relevant

One month post-surgery, patients reported more pain (25 points), dyspnoea (25 points), appetite loss (18 points) and constipation (12 points) on the QLQ-C30 questionnaire compared with baseline (Table 2; Fig. 3). By 3 months, problems with pain, appetite loss and constipation had resolved to baseline levels, though problems with dyspnoea (22 points) were still present at 12 months. QLQ-LC13 scores also indicated increased levels of dyspnoea compared with baseline at all follow-up time points, which had not resolved 12 months post-operatively (15 points; Table 2; Fig. 4). Problems with chest and other pain measured by the QLQ-LC13 had also increased 1 month after surgery compared with baseline (22 and 17 points, respectively). While problems with other pain had resolved to pre-surgery levels by 3 months post-surgery, problems with chest pain were still present (11 points).

#### Fatigue

**Fig. 5:**
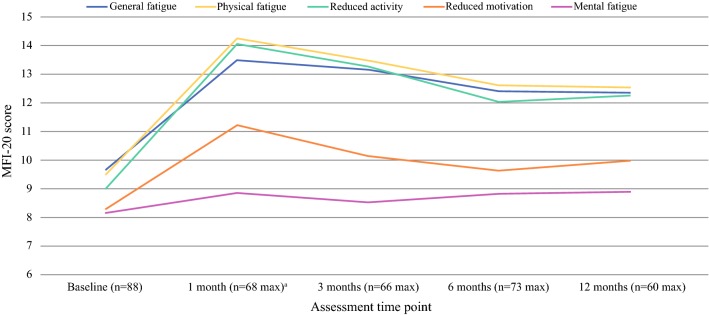
Mean MFI-20 cumulative fatigue scores for patients undergoing VATS. Higher scores for fatigue dimensions suggest an increase in sub-scales of fatigue (general, physical and mental) and increased reduction in activity and motivation. A variable that scored at least 2 points greater or less than the baseline score is considered a clinically important difference

VATS had the greatest impact on patients’ levels of fatigue compared with any other symptom or function of HRQL. One month after surgery, patients reported a 26-point increase in QLQ-C30 fatigue scores compared with baseline (Table 2; Fig. 3). Patients’ fatigue scores on four of the five MFI-20 dimensions (general fatigue, physical fatigue, reduced activity and reduced motivation) had also increased by between 2.9 and 5.0 points compared with baseline, though levels of mental fatigue remained similar (Table 2; Fig. 5). While problems with reduced motivation had recovered to pre-surgery levels by 3 months, patients reported persistent problems with reduced activity (3.2-point difference) and general (2.7 points) and physical fatigue (3 points) on the MFI-20 that were still present 12 months after surgery. EORTC QLQ-C30 scores also showed that clinically significant increases in patients’ problems with fatigue had not resolved during the first year post-surgery.

Post hoc paired *t*-tests comparing mean scores between baseline and expected worse (1 month), early (3 month) and longer-term (12 months) recovery time points showed that all clinically significant differences in function, symptom and fatigue scores observed (described above) were statistically significant at the 1% significance level (*P* < 0.01 for all, Table 2).

## Discussion

This prospective cohort study describes recovery and impact of surgery on HRQL in patients in the first year after VATS lung resection for NSCLC. Patient-reported HRQL assessment using established and validated generic and disease-specific instruments at multiple time points provides a detailed understanding of patients’ recovery after VATS resections to be determined. Patients selected for lung resection by VATS reported significant worsening of several symptoms and reduction in many aspects of HRQL 1 month after surgery. While many problems had resolved by 3 months post-surgery, patients reported significant ongoing reductions in physical, role and social function, and persistent fatigue and dyspnoea that had still not recovered 12 months post-surgery.

This study also demonstrates that HRQL data collection in patients undergoing surgery for NSCLC is possible. Questionnaire response rates and levels of data completeness in the present study were high at all assessment time points, and participant withdrawals infrequent. The study demonstrates that HRQL data can be collected comprehensively in future trials.

The present work indicates that the use of VATS approaches to lung cancer resection instead of open surgery does not prevent significant and prolonged HRQL changes, and that the detrimental impact of VATS lung resection on the HRQL of patients with NSCLC may be more extensive and prolonged than previously thought. Symptoms of dyspnoea and fatigue, in particular, persisted to the end of follow-up, highlighting areas where future interventions to improve HRQL might be directed. This finding contrasts with earlier studies.^10^,^11^,^14^ Bendixen and colleagues for example described self-reported HRQL of VATS lobectomy patients as high during 12 months of follow-up, though this was assessed by the generic EuroQol EQ-5D-3L questionnaire, fatigue and lung cancer-specific HRQL were not evaluated in detail, and response rates and data completeness were low.^14^

Established and validated patient-reported outcome measures were used to measure HRQL in this study, and questionnaire response rates and levels of data completeness were high. The present work, however, is a single-centre prospective cohort study, with patients selected for surgery by thoracic surgeons working within lung cancer multidisciplinary teams, and consequently, it is possible that the characteristics of the patient sample included do not reflect those of the wider population of patients undergoing VATS for NSCLC. This study included patients diagnosed with stage IA–IV lung cancer and patients who underwent surgery for locally advanced or oligometastatic NSCLC. In accordance with current practice, a minority (approximately 30% of all VATS patients in this study) underwent adjuvant chemotherapy after surgery. Literature indicates that post-operative chemotherapy has a significant impact on patients’ HRQL,^27^ and this should be considered when interpreting the study findings. Patients whose surgery was converted from VATS to open or whose diagnosis changed (e.g. to a benign diagnosis) following pathological assessment were excluded. Two patients were converted from VATS to open surgery due to major bleeding, approximating the 5% conversion rate reported in national audit data.^1^ The analysis therefore describes HRQL only in successfully completed VATS cases. By using this approach, the impact on HRQL seen after VATS surgery is independent of the conversion rate. Since one of the main objectives of the present work is to inform future randomised trial designs, and the conversion rate might be expected to change over time, this was felt to be reasonable.

Patients with benign conditions were excluded because it was hypothesised that HRQL may be impacted differently for patients undergoing surgery for benign conditions. Future studies may wish to explore possible differences in HRQL between patients undergoing surgery for benign and malignant conditions. Larger studies are also needed to study subgroups accurately and in detail for example patients undergoing VATS surgery followed by adjuvant chemotherapy or patients undergoing sub-lobar lung resections. The age and gender of the included participants are, however, broadly similar to those included in the study by Bendixen et al.,^14^ although the latter included patients with early-stage disease only. Comparisons (*t*-tests) between HRQL scores at baseline and follow-up time points were undertaken post hoc, and the sample size was not specifically powered to accurately and reliably detect meaningful differences in HRQL scores between time points. It is possible, therefore, that this may have resulted in false-negative findings (type II error). In addition, though a significance criterion of 1% was used throughout, it is possible that multiple significance testing may have resulted in false-positive findings (type I error).

While assessment of the HRQL impact of open surgery was planned a priori, the small number of participants receiving open surgery meant that accurate assessment was not possible. Baseline patient characteristics indicated that patients undergoing VATS surgery were more often diagnosed pre-operatively with lower-stage tumours, had a better Thoracoscore, were more likely to be older and current or recent smoker and less likely to be obese than patients undergoing open surgery. The open group also included a greater proportion of pneumonectomies compared with the VATS group. Literature suggests that pneumonectomies are associated with inferior post-operative HRQL.^11^ This shows how participants were selected differently for each procedure. Consequently, a post hoc decision was taken not to evaluate the HRQL data in this group, to avoid unreliable comparisons with the VATS group regarding HRQL that may result in misleading conclusions being drawn from the data. Summary data describing the baseline demographic and clinical characteristics, and post-operative and 12-month follow-up clinical outcomes are, however, still presented for transparency and to make available information about all participants recruited for this study that may be of interest to the reader to interpret the study findings and design future studies. These data may also be of use to inform the design of future studies in this field. Participant numbers were also too small to enable an accurate comparative assessment of recovery and HRQL according to the magnitude of VATS resection performed (e.g. to compare patients undergoing lobectomy or sub-lobar resections). Nevertheless, the data in this manuscript provide a foundation for future work in a larger cohort of patients to explore the impact of the magnitude of resections on patients’ HRQL. Further work to evaluate HRQL in a larger sample of participants undergoing VATS and open surgery is currently taking place in the ongoing multicentre VIOLET randomised trial (ISRCTN13472721).^28^

Assessment of patient-reported HRQL can enable in-depth understanding of patients’ experiences of recovery after VATS for NSCLC that is critical to promote patient-centred care and guide clinical decision-making alongside clinical and survival data.^29^,^30^ Rigorous assessment of HRQL using validated and multidimensional outcome measurement instruments is also central to providing patients with accurate and detailed information about expected recovery and impact on HRQL and the process of fully informed consent for surgery.^30^ Clinicians may consider communicating this information in discussions with patients prior to surgery.

## Electronic supplementary material

Below is the link to the electronic supplementary material.

Supplementary material 1 (DOCX 42 kb)
