# Supplementary material for: Impact on Health-Related Quality of Life of Video-Assisted Thoracoscopic Surgery for Lung Cancer
Source: Ann Surg Oncol. 2019 Dec 1;27(4):1259–71. doi: 10.1245/s10434-019-08090-4 (PMC7060150; doi:10.1245/s10434-019-08090-4)
Supplement: Supplementary file 1 — Supplementary material 1 (DOCX 42 kb) [file 10434_2019_8090_MOESM1_ESM.docx]

**Supplementary Table 1.** Baseline demographic and clinical characteristics of patients undergoing VATS or open surgery (n=110)

|  | **Surgical procedure received** | | |
| --- | --- | --- | --- |
| **Variable** | **VATS (n=92)** | **Open (n=18)** | **All (n=110)** |
| **Age categories** | **92** | **18** | **110** |
| <60 years | 13 (14.1) | 4 (22.2) | 17 (15.5) |
| 60-69 years | 29 (31.5) | 10 (55.6) | 39 (35.5) |
| 70-79 years | 35 (38.0) | 3 (16.7) | 38 (34.5) |
| ≥80 years | 15 (16.3) | 1 (5.6) | 16 (14.5) |
| Mean (SD) age in years | 70.3 (8.8) | 65.1 (8.8) | 69.4 (9.0) |
| **Men** | 53 (57.6) | 11 (61.1) | 64 (58.2) |
| **Clinical stage ^a^:** | **92** | **18** | **110** |
| IA | 31 (33.7) | 0 | 31 (28.2) |
| IB | 22 (23.9) | 2 (11.1) | 24 (21.8) |
| IIA | 13 (14.1) | 4 (22.2) | 17 (15.5) |
| IIB | 9 (9.8) | 6 (33.3) | 15 (13.6) |
| IIIA | 15 (16.3) | 4 (22.2) | 19 (17.3) |
| IIIB | 0 (0.0) | 2 (11.1) | 2 (1.8) |
| IV | 2 (2.2) | 0 | 2 (1.8) |
| **Thoracoscore ^b^** | **92** | **18** | **110** |
| Median (IQR) | 1.5 (1.2, 1.9) | 2.35 (1.8, 4.5) | 1.9 (1.2, 2.3) |
| **MRC dyspnoea grade ^c^** | **92** | **17** | **109** |
| 1 | 37 (40.2) | 5 (27.9) | 42 (38.2) |
| 2 | 40 (43.5) | 8 (44.4) | 48 (43.6) |
| 3 | 14 (15.2) | 3 (16.7) | 17 (15.5) |
| 4 | 1 (1.1) | 1 (5.6) | 2 (1.8) |
| 5 | 0 | 0 | 0 |
| Missing | 0 | 1 (5.6) | 1 (0.9) |
| **WHO performance score** ^d^ | **91** | **18** | **109** |
| 0 | 37 (40.2) | 6 (33.3) | 43 (39.1) |
| 1 | 44 (47.8) | 7 (38.9) | 51 (46.4) |
| 2 | 10 (10.9) | 3 (16.7) | 13 (11.8) |
| 3 | 0 | 1 (5.6) | 1 (0.9) |
| 4 | 0 | 1 (5.6) | 1 (0.9) |
| Missing | 1 (1.1) | 0 | 1 (0.9) |
| **Predicted FEV_1_** | **92** | **17** | **109** |
| ≥80% | 55 (59.8) | 8 (44.4) | 63 (57.8) |
| 60-79% | 28 (30.4) | 5 (27.8) | 33 (30.3) |
| 40-59% | 9 (9.8) | 2 (11.1) | 11 (10.1) |
| <40% | 0 | 2 (11.1) | 2 (1.8) |
| Missing | 0 | 1 (5.6) | 1 (0.9) |
| **Predicted FVC** | **91** | **17** | **108** |
| Median (IQR) | 103 (86, 119) | 95 (89, 107) | 101 (86.5, 114) |
| Missing | 1 (1.1) | 1 (5.6) | 2 (1.8) |
| **Planned surgical procedure ^e^** | **88** | **18** | **106** |
| Segmental resection | 5 (5.4) | 0 | 5 (4.5) |
| Wedge resection | 12 (13.0) | 0 | 12 (10.9) |
| Lobectomy and bi-lobectomy | 71 (77.2) | 11 (61.1) | 88 (83.0) |
| Pneumonectomy | 0 | 7 (38.9) | 7 (6.6) |
| Missing | 4 (4.3) | 0 | 4 (3.8) |
| **BMI categories ^f^** | **92** | **18** | **110** |
| Underweight (<18.5) | 2 (2.2) | 0 | 2 (1.8) |
| Normal (18.5-24.9) | 25 (27.2) | 3 (16.7) | 28 (25.5) |
| Overweight (25-29.9) | 35 (38.0) | 5 (27.8) | 40 (36.4) |
| Clinically obese (30-39.9) | 28 (30.4) | 8 (44.4) | 36 (32.7) |
| Morbidly obese (≥40) | 2 (2.2) | 2 (11.1) | 4 (3.6) |
| **Smoking status:** | **92** | **18** | **110** |
| Current smoker | 19 (20.7) | 0 | 19 (17.3) |
| Gave up < 6 weeks ago | 10 (10.9) | 1 (5.5) | 11 (10.0) |
| Gave up > 6 weeks ago | 21 (22.8) | 4 (22.2) | 25 (22.7) |
| Gave up > 1 year ago | 34 (37.0) | 11 (61.1) | 45 (40.9) |
| Never smoked | 8 (8.7) | 2 (11.1) | 10 (9.1) |
| **Employment status:** | **92** | **18** | **110** |
| Employed | 24 (26.1) | 4 (22.2) | 28 (25.5) |
| Unemployed | 1 (1.1) | 0 | 1 (0.9) |
| Retired | 63 (68.5) | 10 (55.5) | 73 (66.4) |
| Other ^g^ | 4 (4.3) | 4 (22.2) | 8 (7.3) |

Values in parentheses are percentages unless indicated otherwise

^a^ Pre-operative clinical staging of tumours ^1^

^b^ Thoracoscore scoring system ^2^

^c^ MRC dyspnoea grading system ^3^

^d^ WHO performance score criteria ^4^

^e^ Decision made by multi-disciplinary team prior to surgery based on available clinical and pathological information.

^f^ BMI category criteria ^5^

^g^ Of the 4 VATS patients, 1 was registered disabled, 1 was on long term sick leave, 1 was a volunteer and 1 was a housewife. Of the 4 open patients, 2 were registered disabled and 2 were housewives.

**Abbreviations:** BMI – body mass index; FEV_1_ – forced expiratory volume in one second; FVC – forced vital capacity; IQR – inter-quartile range; MRC - Medical Research Council; SD – standard deviation; VATS – video-assisted thoracoscopic surgery; WHO – World Health Organization

**Supplementary Table 2.** Post-operative clinical outcomes

|  | **Surgical procedure received** | | |
| --- | --- | --- | --- |
|  | **VATS** (n=92) | **Open** (n=18) | **All** (n=110) |
| **Surgical procedure performed** | **92** | **18** | **110** |
| Segmental resection | 10 (10.9) | 0 | 10 (9.1) |
| Wedge resection | 13 (14.1) | 0 | 13 (11.8) |
| Lobectomy | 69 (75.0) | 12 (66.7) | 81 (73.6) |
| Pneumonectomy | 0 | 6 (33.3) | 6 (5.5) |
| **Epidural anaesthesia used peri-operatively** | **92** | **18** | **110** |
| Yes | 0 | 1 (5.6) | 1 (0.9) |
| **Paravertebral anaesthesia used peri-operatively** | **92** | **18** | **110** |
| Yes | 89 (96.7) | 16 (88.9) | 105 (95.5) |
| **Returned to theatre during same admission** | **92** | **17** | **109** |
| Yes | 5 (5.4) | 2 (11.1) | 7 (6.4) |
| Missing | 0 | 1 (5.6) | 1 (0.9) |
| **Ventilated on ITU any time post-operatively** | **92** | **17** | **109** |
| Yes | 3 (3.3) | 3 (16.7) | 6 (5.5) |
| Missing | 0 | 1 (5.6) | 1 (0.9) |
| **Air leak for > 7 days post-operatively** | **92** | **17** | **109** |
| Yes | 16 (17.4) | 2 (11.1) | 18 (16.4) |
| Missing | 0 | 1 (5.6) | 1 (0.9) |
| **Infection requiring extension of in-hospital stay** | **92** | **17** | **109** |
| Yes | 8 (8.7) | 6 (33.3) | 14 (12.7) |
| Missing | 0 | 1 (5.6) | 1 (0.9) |
| **Unplanned admission or readmission in to ITU/HDU** | **92** | **17** | **109** |
| Yes | 3 (3.3) | 3 (16.7) | 6 (5.5) |
| Missing | 0 | 1 (5.6) | 1 (0.9) |
| **Length of hospital stay, days** | **91^a^** | **18** | **107** |
| Median (IQR) | 5 (3, 7) | 6 (5, 11) | 5 (4, 8) |
| **Pathological tumour stage ^b^:** | **92** | **18** | **110** |
| IA | 34 (37.0) | 1 (5.6) | 35 (31.8) |
| IB | 28 (30.4) | 2 (11.1) | 30 (27.3) |
| IIA | 7 (7.6) | 3 (16.7) | 10 (9.1) |
| IIB | 10 (10.9) | 6 (33.3) | 16 (14.5) |
| IIIA | 10 (10.9) | 6 (33.3) | 16 (14.5) |
| IIIB | 0 | 0 | 0 |
| IV | 3 (3.3) | 0 | 3 (2.7) |

^a^ One patient died in hospital two days post-operatively and was therefore not included in the analysis of length of hospital stay

^b^ Post-operative clinical staging of tumours ^1^

**Abbreviations:** HDU – high dependency unit; IQR – inter-quartile range; ITU – intensive therapy unit; SD – standard deviation; VATS – video-assisted thoracoscopic surgery

**Supplementary Table 3.** 12-month follow-up clinical outcomes

|  | **Surgical procedure received** | | |
| --- | --- | --- | --- |
| **Variable** | **VATS** (n=92) | **Open** (n=18) | **All** (n=110) |
| **Did the patient receive any further intervention for cancer?** | **92** | **17** | **109** |
| Yes | 27 (29.3) | 10 (55.5) | 37 (33.6) |
| If yes, what treatment did the patient have? |  |  |  |
| Radiotherapy alone | 6 (22.2) | 1 (10.0) | 7 (18.9) |
| Chemotherapy alone | 12 (44.4) | 5 (50.0) | 17 (45.9) |
| Radiotherapy and chemotherapy | 3 (11.1) | 1 (10.0) | 4 (10.8) |
| Surgery | 1 (3.7) | 0 | 1 (2.7) |
| Palliative care | 2 (7.4) | 1 (10.0) | 3 (8.1) |
| Chemotherapy and palliative care | 1 (3.7) | 2 (20.0) | 3 (8.1) |
| Missing | 2 (7.4) | 0 | 2 (5.4) |
| Missing | 0 | 1 (5.6) | 1 (0.9) |
| **Was the patient still alive at 12 months?** | **92** | **18** | **110** |
| Yes | 82 (89.1) | 12 (66.7) | 94 (85.5) |
| If yes, was there disease recurrence? |  |  |  |
| Yes | 14 (17.1) | 3 (25.0) | 17 (18.1) |
| No | 10 (10.9) | 6 (33.3) | 16 (14.5) |
| If no, was there disease recurrence before the patient died? |  |  |  |
| Yes | 5 (50.0) | 1 (16.7) | 6 (37.5) |
| Not applicable ^a^ | 3 (30.0) | 3 (50.0) | 6 (37.5) |
| **Cumulative death within 12-month follow-up period** | **92** | **18** | **110** |
| Death within 1 month | 3 (3.3) | 0 | 3 (2.7) |
| Death within 3 months | 3 (3.3) | 2 (11.1) | 5 (4.5) |
| Death within 12 months | 10 (10.9) | 6 (33.3) | 16 (14.5) |

^a^ Patients did not survive for long enough post-surgery for disease recurrence data to be collected

**Abbreviation:** VATS – video-assisted thoracoscopic surgery

**References**

1. Brierley JD, Gospodarowicz MK, Wittekind C. *TNM Classification of Malignant Tumours*. 8th edition.Wiley-Blackwell; 2017.

2. Falcoz PE, Conti M, Brouchet L, et al. The Thoracic Surgery Scoring System (Thoracoscore): Risk model for in-hospital death in 15,183 patients requiring thoracic surgery. *J Thorac Cardiovasc Surg*. 2007;133 (2):325-332.e321. <https://doi.org/10.1016/j.jtcvs.2006.09.020>

3. Fletcher CM. The clinical diagnosis of pulmonary emphysema; an experimental study. *Proc R Soc Med*. 1952;45 (9):576-586.

4. Oken MM, Creech RH, Tormey DC, et al. Toxicity and response criteria of the Eastern Cooperative Oncology Group. *Am J Clin Oncol*. 1982;5 (6):649-655.

5. Anonymous. Physical status: the use and interpretation of anthropometry. Report of a WHO Expert Committee. *World Health Organ Tech Rep Ser*. 1995;854 1-452.
